# Unraveling the Enigma of Managing a Large Periapical Lesion: A Case Report

**DOI:** 10.7759/cureus.53031

**Published:** 2024-01-27

**Authors:** Mrinal Nadgouda, Aditya Patel, Manoj Chandak, Anuja Ikhar, Swayangprabha Sarangi

**Affiliations:** 1 Conservative Dentistry and Endodontics, Sharad Pawar Dental College and Hospital, Datta Meghe Institute of Higher Education and Research, Wardha, IND

**Keywords:** screw, miniplate, odontogenic tumor, surgical endodontics, apicoectomy

## Abstract

This case report documents the diagnosis and successful management of a substantial periapical lesion located in the lower left region of the jaw. The patient presented with clinical symptoms indicative of periapical pathology, and radiographic examination revealed an extensive radiolucent lesion. The chosen treatment approach involved endodontic intervention coupled with surgical decompression, leading to the resolution of the lesion and restoration of oral health. This case underscores the significance of an accurate diagnosis and a multidisciplinary treatment approach in addressing large periapical lesions.

## Introduction

Periapical lesions, often arising from pulpal infections, may appear as radiolucent regions on radiographic images. While conventional endodontic therapy is effective in many cases, the management of large lesions can present challenges, necessitating a comprehensive treatment approach. This case study highlights a patient with a significant periapical lesion in the lower left jaw region, underscoring the importance of a meticulous diagnostic process and an integrated treatment plan. Radicular cysts stand out among the 52-68% cystic lesions affecting jaws. Typically located at the apices of teeth that are affected, they also have the ability to reach onto the lateral aspects of the roots in association with accessory canals [1]. Radicular cysts are believed to originate from the epithelial cell rests of Malassez and remnants of Hertwig's epithelial root sheath present in the periodontal ligament. In conditions of pulpal inflammation, triggers arise from the region of inflammation, resulting in the expansion of these epithelial cells localized in the periodontal ligament [2]. Conservative management through nonsurgical endodontic therapy is frequently suitable for smaller periapical cysts. Proper endodontic treatment removes irritants from the canals through chemomechanical instrumentation. The complete sealing of the root canal results in the gradual resolution of all cell components involved in the inflammatory reaction [3]. Radicular cysts develop as a result of persistently inflamed granulation tissue, commonly known as periapical granuloma, situated near the apex of teeth, regardless of whether they have undergone endodontic treatment with an infected root canal system.

Research conducted by Oztan and Kalaskar et al. has confirmed that substantial periapical lesions, encompassing cysts, can exhibit favorable responses to nonsurgical treatment utilizing calcium hydroxide paste [4]. Nevertheless, when root canal treatment proves impractical or unsuccessful, periapical surgery emerges as a viable and predictable alternative. In a retrospective observational study conducted by Hyun-Kyung et al., enucleation with apicectomy was identified as the predominant management approach for radicular cysts [5]. This case report underscores the diagnosis and successful surgical management of a substantial periapical cyst in the lower left posterior jaw region.

## Case presentation

**Figure 1:**
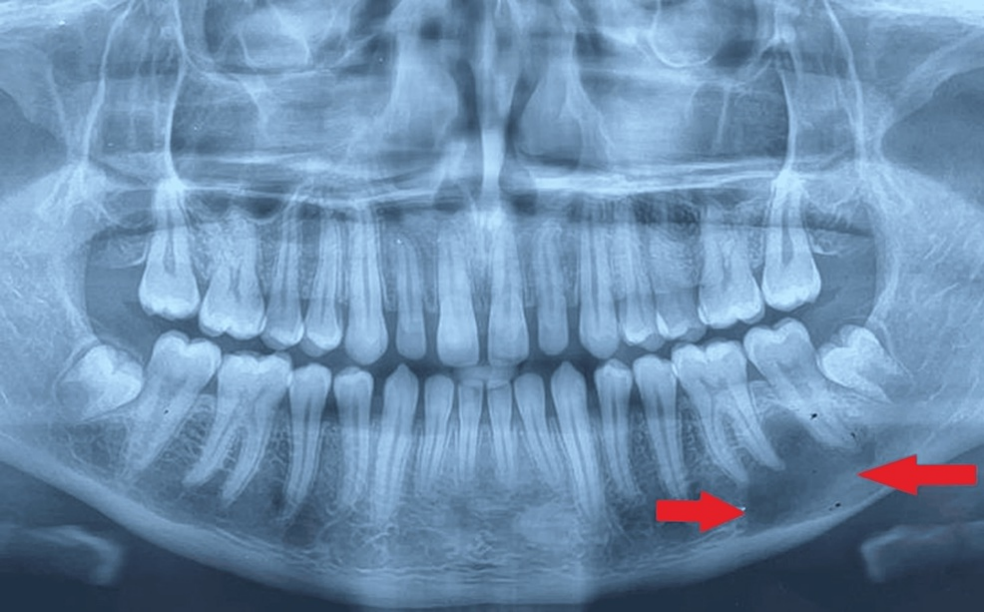
Preoperative orthopantomogram indicating expansion of the periapical pathology in relation to teeth 36 and 37.

A 22-year-old male patient presented to the Orthodontics Department with a chief complaint of malaligned teeth. The vitals of the patient, including past medical history, past dental history, and drug history, revealed no evidence of the presence of systemic hypertension, diabetes mellitus, or other chronic illnesses. However, a radiographic assessment using an orthopantomogram revealed a sizable periapical pathology associated with teeth 36 and 37 (Figure 1).

**Figure 2:**
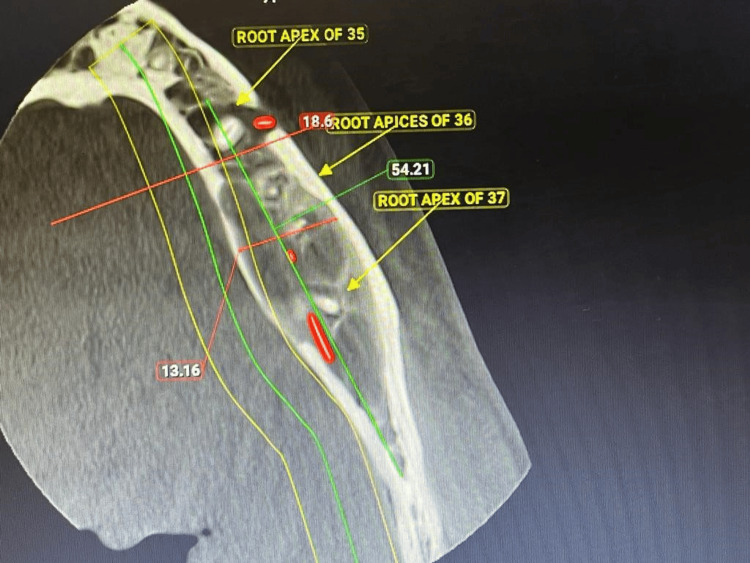
CBCT of the lower right mandibular area showing a buccolingual expansion of bone at the region pertaining to teeth 36 and 37. CBCT: Cone beam computed tomography

Consequently, the patient was referred to the Department of Conservative Dentistry and Endodontics for further evaluation. Clinical examination indicated no evidence of caries affecting the above-mentioned teeth. The teeth were completely asymptomatic as they showed no symptoms of night pain, fever, or history of trauma. Intraoral examination revealed no tenderness on percussion with teeth 36 and 37, but slight swelling was observed at the level of attached gingiva with no evidence of a periodontal pocket. Pulp sensibility tests indicated no response with teeth 36 and 37, while adjacent teeth exhibited a normal response. Subsequently, a cone beam computed tomography was recommended to assess the dimensions of the lesion further. The imaging revealed a substantial radiolucent area measuring 3 x 2 cm, with a length of 32.95 mm and a width of 18.42 mm, associated with teeth 36 and 37. Thinning of the lingual cortical plate was evident, along with an expansion of the buccal and lingual cortical plates, showcasing an increase in the anteroposterior dimension compared to buccolingual expansion. Importantly, no downward displacement of the inferior alveolar canal or compression of the inferior alveolar nerve was observed (Figure 2).

After a thorough histological examination, the final confirmation of the lesion pertaining to the Keratocystic odontogenic tumor was concluded with teeth 36 and 37. The patient was explained about the procedure and informed consent was obtained prior to the commencement of the treatment.

Treatment

Endodontic Phase

**Figure 3:**
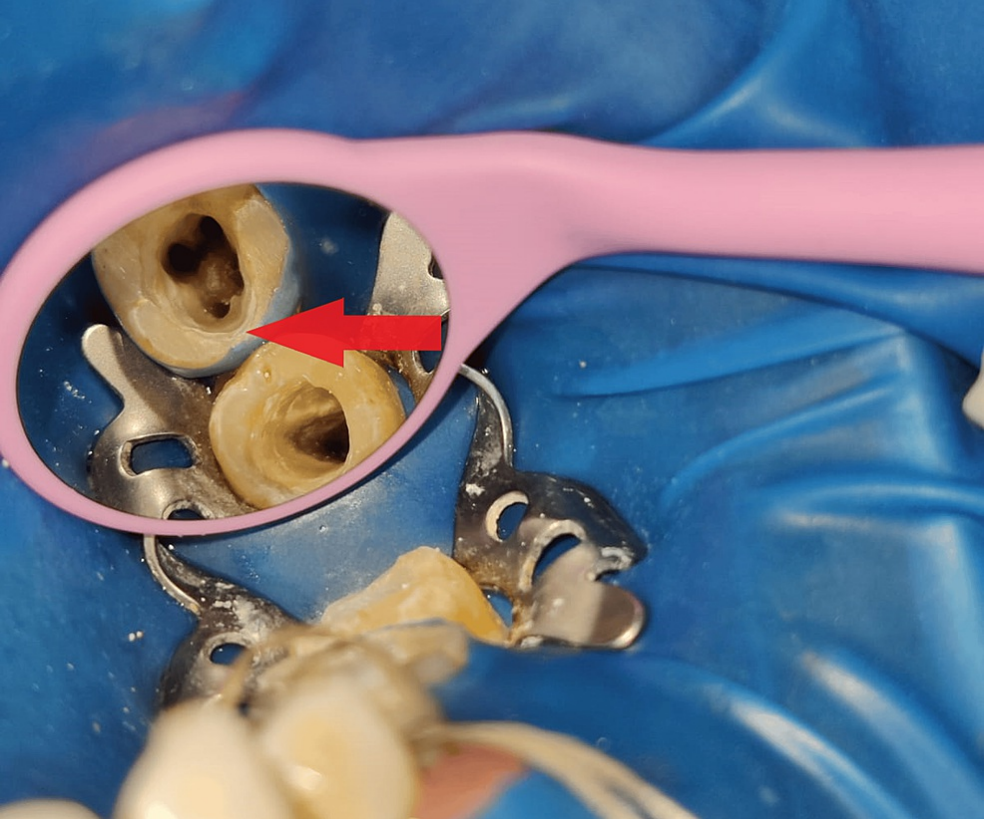
Rubber dam isolation and access opening done with teeth 36 and 37.

Local anesthesia with 1:100,000 epinephrine was administered, followed by the placement of a rubber dam for isolation with teeth 36 and 37. Access opening was performed using a BR-45 round bur and a safe-end bur (EX-24 Mani, Japan) (Figure 3).

Patency filing was carried out and the working length was recorded using an apex locator (J.W. Morita, Japan) which was subsequently confirmed radiographically. Biomechanical preparation was done up to the F3 ProTaper (20/.06) (Neoendo flex, Orikam Healthcare Private Limited, India) with teeth 36 and 37.

**Figure 4:**
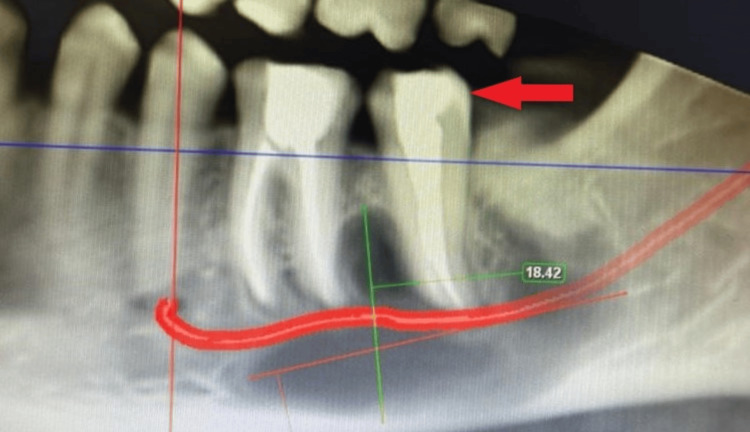
CBCT evaluation following endodontic treatment of teeth 36 and 37. CBCT: Cone beam computed tomography

Calcium Hydroxide intracanal medicament (RC Cal, Prime Dental, India) was placed followed by placement of a temporary restoration (Cavit, 3M ESPE, Germany) in teeth 36 and 37 and the patient was recalled after one week for follow-up. A temporary dressing was removed during the second visit and the canal was irrigated using normal saline. Ultrasonic activation in the canal space was done to remove calcium hydroxide using an endoactivator (Dentsply Sirona, USA) and a repeat dressing of calcium hydroxide medicament (RC Cal, Prime Dental, India) was given. After a span of about one week, the patient was again recalled for the third visit where it was found that the patient was completely asymptomatic with negligible presence of any symptoms in association with the lower teeth. Obturation of the radicular space was done followed by placement of permanent composite resin (Spectrum, Dentsply Sirona, USA), which was confirmed using a cone beam computed tomography in teeth 36 and 37 (Figure 4).

Surgical Phase

**Figure 5:**
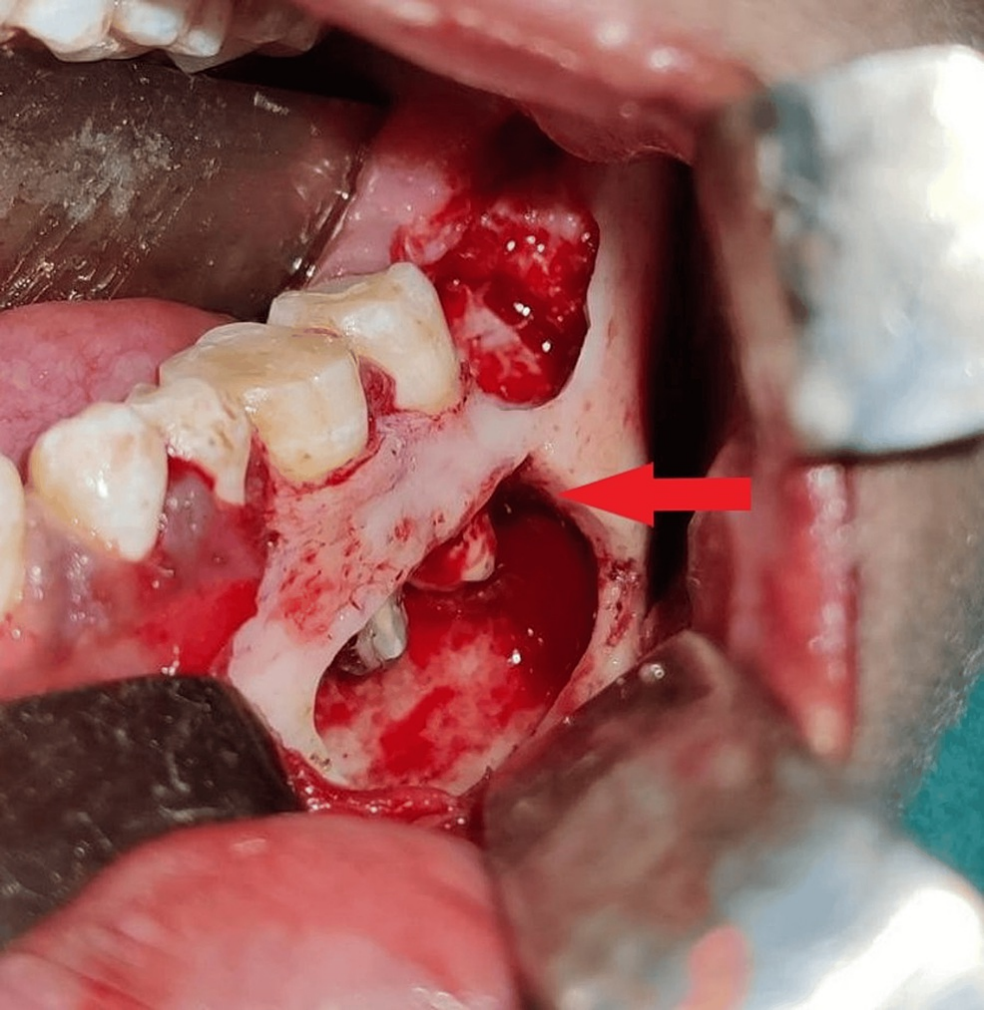
Reflection of full-thickness mucoperiosteal flap showing cystic enucleation, apical resection of teeth 36 and 37 followed by placement of Biodentine.

The surgical phase of the treatment comprised a prior assessment of hematological parameters (complete blood count, haemoglobin, bleeding time, clotting time, random blood sugar and partial prothrombin time) after which the patient was considered fit for surgery and was scheduled for a surgical cystic enucleation treatment protocol. A crevicular incision along with a vertical releasing incision was given from teeth 36 to 37 using BP blade number 15, a full-thickness mucoperiosteal flap was then raised and the root apex of teeth 36, 37 was exposed. A bony window was created, enucleation was done using surgical spoon curettes (GDC, Hoshiarpur, India) with teeth 36, 37 whose apices were then resected 3mm and a retrograde cavity was prepared using round carbide bur (RA 023). A retrograde filling using Biodentine™ (Septodont, France) was placed as restorative material of 3mm thickness (Figure 5).

**Figure 6:**
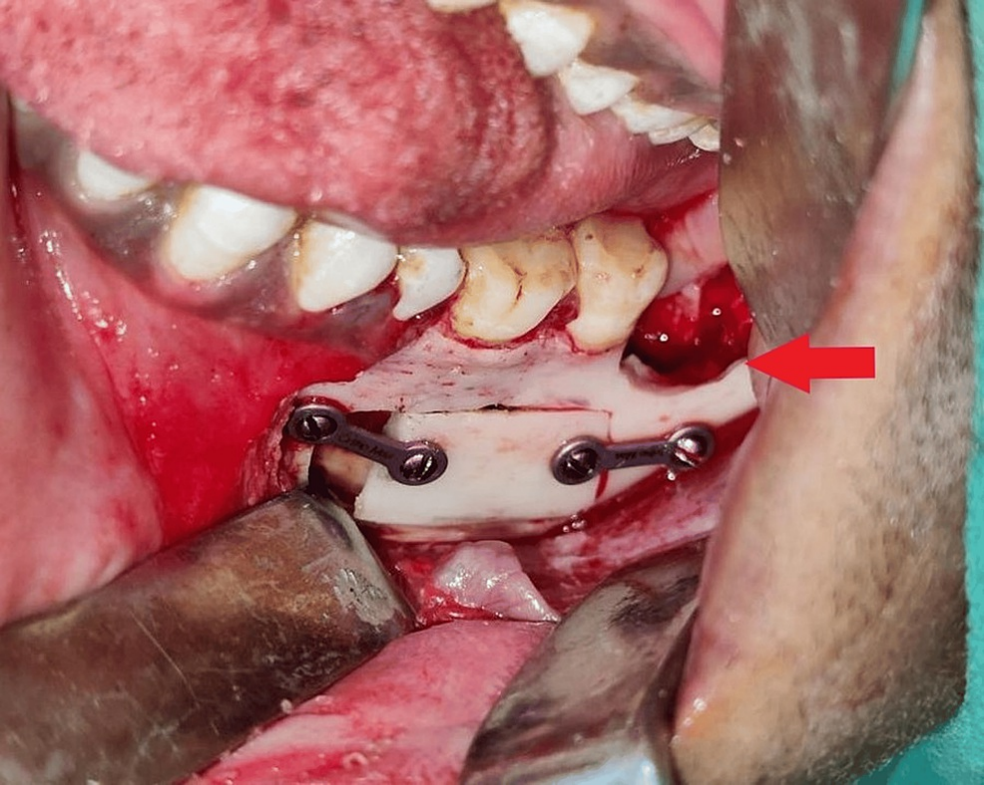
Transalveolar extraction done with tooth 38 and bone plating with the placement of mini-screws in the 36 and 37 region.

**Figure 7:**
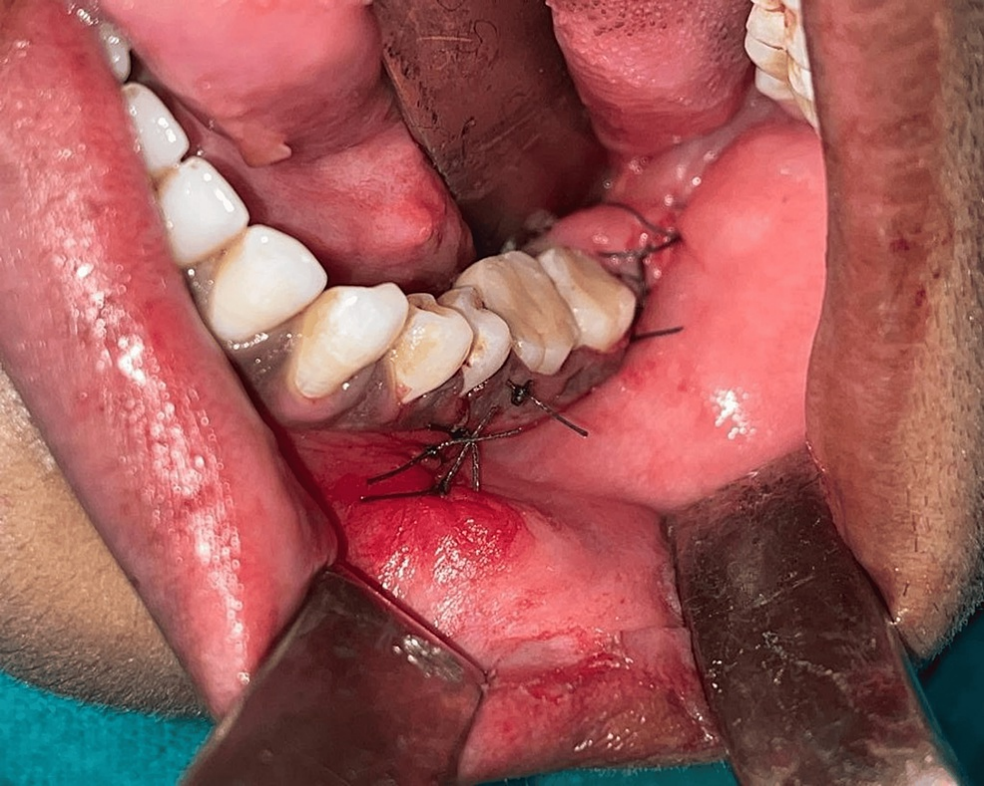
Suturing of the reflected flap

A transalveolar extraction was performed with 38 and bone plating was done using a miniplate and screws followed by suturing of the reflected flap (Figures 6, 7).

**Figure 8:**
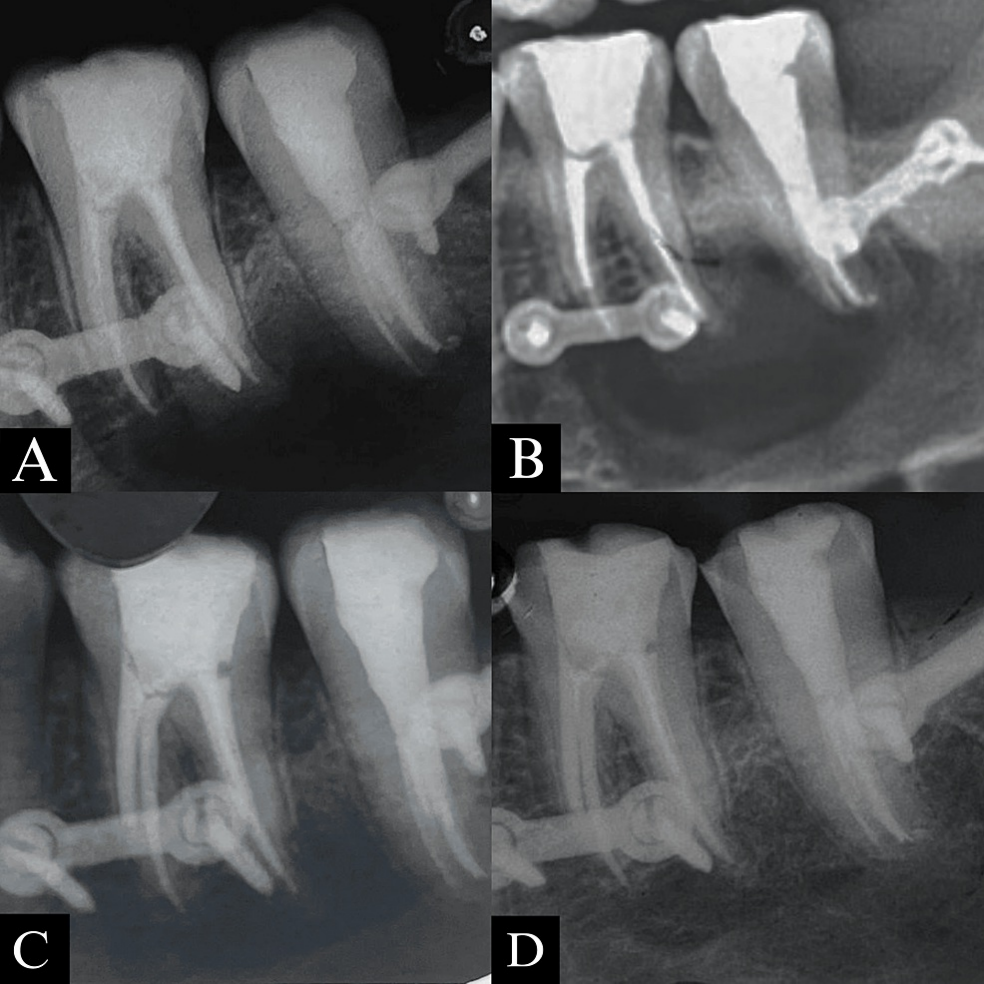
(A) Immediate post-operative IOPA; (B) Three months follow-up IOPA; (C) Six months follow-up IOPA; (D) One year follow-up IOPA. IOPA: Intraoral periapical radiograph

The patient reported to the Department of Conservative Dentistry after 3, 6 and 12 months for follow-up and a considerable amount of healing and bone growth was seen in the area pertaining to teeth 36 and 37 (Figure 8).

## Discussion

Odontogenic keratocysts (OKCs) are developmental odontogenic cysts known for their potential aggressive growth and local invasion [6]. When an OKC affects the periapical region of a tooth, it presents a unique challenge to traditional endodontic management. In instances where conventional root canal therapy proves insufficient or impractical, apicectomy becomes a valuable adjunctive procedure. This discussion explores the rationale, challenges, and outcomes associated with performing apicectomy in the context of a large OKC within the mandible.

Large OKCs may exhibit a high recurrence rate, particularly if not adequately treated [7]. Apicectomy facilitates the removal of any residual cystic lining along with its contents near the affected tooth's apex, reducing the risk of recurrence. In cases where the affected tooth is vital and holds functional significance, apicectomy offers an opportunity to preserve the tooth instead of resorting to extraction [8]. The preservation of natural dentition is advantageous for both functional and aesthetic reasons. Apicectomy enables a more extensive and precise sampling of periapical tissues for histopathological examination, which is crucial for confirming the lesion's nature and ensuring thorough excision of all pathological tissue. Large OKCs may extend beyond the periapical region, posing a challenge for complete removal of the cystic lining through conventional apical surgery [9]. A meticulous and comprehensive approach is necessary to minimize the risk of recurrence.

The mandible harbors critical anatomical structures like nerves and blood vessels, emphasizing the importance of surgical precision to avoid damage and minimize postoperative complications [10]. While apicectomy addresses the periapical component of the lesion, the potential for recurrence persists. A comprehensive treatment plan, often involving enucleation of the cystic lesion and vigilant monitoring, is crucial to mitigate this risk [11]. The excised tissue undergoes a thorough histopathological examination to confirm the OKC diagnosis and assess margins for complete removal, aiding in determining the surgical intervention's success. Patients receive clear postoperative care instructions, and regular follow-up appointments are essential to monitor healing, detect any signs of recurrence, and promptly address complications. In some cases, depending on the lesion's size and nature, adjunctive therapies like chemical cauterization or adjuvant treatments may be considered to enhance the overall success of the intervention [6].

## Conclusions

Apicectomy plays a crucial role in the management of large OKCs involving the mandible. Despite the challenges associated with the procedure, the potential benefits, including the preservation of the affected tooth and a reduction in recurrence risk, make it a valuable therapeutic option. Careful case selection, precise surgical technique, and a multidisciplinary approach contribute to successful outcomes in addressing these complex lesions. Long-term follow-up remains essential to ensure sustained treatment success and monitor for any signs of recurrence.
